# Conjunctiva Resident γδ T Cells Expressed High Level of IL-17A and Promoted the Severity of Dry Eye

**DOI:** 10.1167/iovs.63.12.13

**Published:** 2022-11-09

**Authors:** Ling Li, Yanxiao Li, Xinhao Zhu, Biao Wu, Zhuo Tang, Han Wen, Jianshu Yuan, Qinxiang Zheng, Wei Chen

**Affiliations:** 1 School of Ophthalmology and Optometry and Eye Hospital, Wenzhou Medical University, Wenzhou, Zhejiang, China; 2 The Affiliated Ningbo Eye Hospital of Wenzhou Medical University, Ningbo, Zhejiang, China; 3 Shaoxing people's hospital, Shaoxing, Zhejiang, China

**Keywords:** γδ T cells, dry eye, IL-17A, conjunctiva

## Abstract

**Purpose:**

Conjunctival inflammation promotes ocular surface disorders in dry eye disease (DED). Here we identified γδ T cells as the predominant source of IL-17A in the murine conjunctiva and assessed their contribution to the pathogenesis of DED.

**Methods:**

We enrolled 22 patients with DED, and analyzed the proportion of γδ T cells in the conjunctival epithelial samples by flow cytometry. Adult C57Bl/6 wild-type and *TCRδ^−^^/^^−^* mice were used to induce DED models to investigate the role of γδ T cells. The characteristics of immune cell infiltration and the expression of immune-related cytokines or markers in mouse conjunctiva were analyzed by flow cytometry, Western blot, and quantitative polymerase chain reaction.

**Results:**

The proportion of γδ T cells in the human DED conjunctiva is significantly higher in patients with severe corneal epithelial defects than in mild ones, which is consistently observed in the murine DED model. Further, a high level of IL-17A but not IFN-γ is detected in the conjunctiva of mice. The increased murine IL-17A–producing cells on the conjunctiva are identified as γδ T cells predominantly and Th17 cells to a lesser extent. Ablation of γδ T cells by antibody depletion or genetic deletion of *TCRδ* alleviates ocular surface damage in the murine DED model.

**Conclusions:**

Our studies evaluate human and experimental murine DED for evidence of γδ T-cell–mediated inflammation and highlight a potential therapeutic synergy by targeting IL-17 and γδ T cells in DED treatment.

Dry eye disease (DED) is a highly prevalent ocular surface autoimmune disorder with a largely unknown pathogenesis.^1^ DED is characterized by a dysregulated immune and inflammatory process that affects the ocular surface.^2^ An uncontrolled severe DED can result in corneal ulceration and scarring, which can cause vision loss.^3^^,^^4^ Currently, there are treatments available for DED that target certain aspects of the disease, such as ocular surface inflammation, but not all individuals experience adequate symptom relief.^5^ Previous studies indicate that when DED occurs or develops there are activation and expansion of IFN-γ–producing CD4^+^ T (Th1) and IL-17A–producing CD4^+^ T (Th17) cells in the murine draining lymph nodes.^6^^,^^7^ The investigation of the “classic” CD4^+^ T cells, especially in the regional draining lymph nodes reveals that both IFN-γ and IL-17A produced by those cells contribute to the corneal barrier disruption.^8^^,^^9^ Meanwhile, recent evidence suggests that the ocular surface, particularly the conjunctiva, may participate in DED progression. Further, the increased levels of IFN-γ and IL-17A have been observed consistently in the ocular surface of both clinical DED^10^^–^^12^ and experimental DED.^7^^,^^9^^,^^13^ The ocular mucosa provides the front line of defense against pathogenic or dangerous factors from the environment.^14^ In addition to their protective role as a physical barrier, the conjunctiva contains diffuse lymphatic tissue and organized follicles to actively participate in immune defense, similar to other mucosal tissues.^15^^,^^16^ Those organized structures resemble the tertiary lymphoid structures^17^ identified in other organs such as the gastrointestinal system,^18^ respiratory system,^19^ special senses,^20^ and hematolymphoid system.^21^ They contain the essential cell types to generate acquired immune responses, including antigen-presenting cells (APCs), B cells, and T cells. Interestingly, cellular composition characterization shows that up to 50% of the immune cells residing in the tertiary lymphoid structure-like structures are γδ T cells.^22^ However, the precise roles of the tertiary lymphoid structure-resident immune cells together with the cellular origin of the locally produced cytokines remain to be identified.

γδ T cells emerge as a crucial immune population in regulating mucosal immunity.^23^^,^^24^ They are neither major histocompatibility complex restricted nor neoantigen dependent, constituting a distinct layer of autoimmune and inflammatory immunity aside from their αβ T-cell counterparts.^25^ Many of the properties of γδ T cells, including the perception of stress-induced changes and very rapid effector responses, are most consistent with innate immunity or lymphoid stress surveillance, although in some cases, they exhibit adaptive behavior.^26^ It is thus proposed that γδ T cells function distinctly from either innate or adaptive immune responses.^27^ According to their expression profiles, γδ T cells can be divided into subsets of either IFN-γ– or IL-17A–producing cells.^28^^,^^29^ Recent research suggests that γδ T cells are major IL-17A producers and have shown their involvement in the early onset of immune activation, such as psoriasis,^30^ autoimmune arthritis,^31^ ischemic brain injury,^32^ infected pancreatitis,^33^ and intraocular neovascularization.^34^ Moreover, the IL-17A–producing γδ T cells in many cases express TCRs containing Vγ4 or Vγ6.^31^^,^^33^^,^^35^^–^^37^ Interestingly, γδ T cells are abundantly distributed in the conjunctiva and highly express IL-17A in response to conjunctival commensals, and of these approximately one-half are Vγ4^+^ subset.^23^ Meanwhile, mice with a loss-of-function RXRα mutation exhibited a 4-fold greater percentage of conjunctival γδ T cells with increased expression of IL-17,^39^ and the development and progression of DED seem to be highly dependent on IL-17A.^9^^,^^13^^,^^38^ However, the contribution of γδ T cells itself in the conjunctiva to the occurrence of DED is not known.

In this study, we found that the proportion and number of γδ T cells increased in the human DED conjunctiva with severe corneal epithelial defects. We confirmed the presence of γδ T cells in the mouse conjunctiva and further characterized their associated cytokines in the DED animal model. We unexpectedly found that, different from lymph nodes where Th17 cells were the major IL-17–producing cells, γδ T cells were the main source of IL-17A in the conjunctiva. We showed that γδ T cells were enriched in the conjunctiva and the majority of γδ T cells in conjunctiva expressed high levels of IL-17A but not IFN-γ. When γδ T cells were depleted by anti-TCRγδ neutralization antibody injection or genetic deletion of TCRδ in mice, the ocular surface damage was mitigated. Collectively, we have elucidated that the γδ T cells in conjunctiva were key sources of IL-17A, which promoted corneal epithelial defects and the severity of DED.

## Methods

### Clinical Subjects

The Institutional Review Board of Wenzhou Medical University approved all procedures involving human subjects in accordance with the Declaration of Helsinki (Ethics Approval Number: 2021-209-K-183-01). Written informed consent was obtained from all subjects before participating in the study. Patients with DED were recruited. DED is diagnosed based on the TFOS DEWS II criteria: (1) an Ocular Surface Disease Index of 13 or greater, (2) a tear-film breakup time (TBUT) of less than 10 seconds; or (3) more than 5 spots of corneal fluorescein staining.^40^ The DED severity grading scheme according to the “Chinese expert consensus on definition and classification of dry eye (2020)” was used. The classification of DED severity is mainly based on the signs of DED. Mild DED shows no obvious signs of ocular surface damage under slit lamp microscopy (corneal fluorescein staining of <5 dots), and a TBUT of 2 or more seconds. Severe DED has a corneal damage range of 2 quadrants and above and/or the corneal fluorescein staining of 30 or more dots, and a TBUT of less than 2 seconds. The cornea has severe staining with confluent stains or corneal filaments. Patients with any of the following conditions were excluded: use of contact lenses, ongoing ocular infection, allergy, autoimmune or inflammatory conditions, recent ocular surgery including refractive surgeries, use of ocular medications including topical agents such as antiglaucoma medication, and subjects with the use of systemic medication known to contribute to DED. A total of 22 subjects were included in the final population.

### Ocular Surface Immune Cells Collection

We chose brush cytology as the sampling technique based on previous reports.^41^ The same ophthalmologist collected the conjunctiva cells every time. Eyes were anesthetized with one drop of 0.5% proxymetacaine hydrochloride (Alcaine, Alcon Laboratories, Fort Worth, TX) for 10 minutes, and then 3 rotations of sterile swabs was rubbed lightly on the palpebral conjunctiva, bulbar conjunctiva, and fornical conjunctiva, respectively. Cells were then detached from the swabs by gentle rotation for 3 minutes in a tube containing RPMI1640 medium (ThermoFisher Scientific, Waltham, MA) supplemented with 10% fetal bovine serum (Gibco, Invitrogen, Waltham, MA) on ice. Three more swabs were performed at the conjunctiva, and the samples were pooled before transport to the laboratory for flow cytometry analysis, which was completed in less than 2 hours after sampling. To avoid intereye correlations, only 1 eye per subject was included in the analysis.^42^ In all cases, the worst eye was chosen for cell collection. The worst eye was defined as the most symptomatic one chosen by the patient or poorer clinical signs as determined by the investigator.

### Animals

C57BL/6J (B6 wild-type [WT]) mice aged 6 to 8 weeks were purchased from Shanghai Jiesijie Experimental Animal Co. Ltd. (Shanghai, China). *TCRδ^−^^/^^−^* mice on the C57BL/6J background were purchased from The Jackson Laboratory (Bar Harbor, ME; Jackson Stock No: 002120). Animal experiments were performed by the Institutional ARVO Statement for the Use of Animals in Ophthalmic and Vision Research (Ethics Approval Number: wydw2021-0064).

### Preparation of Single-Cell Suspension From Conjunctiva and Lymph Nodes

Conjunctiva were harvested by excising the palpebral and bulbar conjunctiva. The specimens were then washed and shaken in phosphate-buffered saline containing 20 mM EDTA at 37°C for 15 minutes. The tissues were then cut into pieces and digested in RPMI1640 medium (ThermoFisher Scientific) containing collagenase Ⅳ (Gibco; 1000 U/mL) at 37°C for 30 minutes. Cervical lymph nodes were isolated, minced, and mechanically ground into single-cell suspensions. Cells from conjunctiva and draining lymph nodes were filtered separately through a 70-µm cell strainer after collagenase or grind treatment. Cells collected by this technique were used for flow cytometry.

### Flow Cytometry Analysis (FACS)

For cell-surface staining, cells were blocked with CD16/32 FcR-block (BioLegend, San Diego, CA) for 5 minutes, and stained with fluorescent dye-conjugated mAb for 30 minutes at 4°C. For intracellular cytokine staining, cells were stimulated for 4 hours with PMA (50 ng/mL; Sigma-Aldrich, St. Louis, MO) and ionomycin calcium salt (500 ng/mL; Sigma-Aldrich) in the presence of brefeldin A (2 µg/mL; Sigma-Aldrich) for 2 hours. Cells were stained for surface markers for 30 minutes, then fixed and permeabilized with the commercial kits (Foxp3/Transcription Factor Staining Buffer; eBioscience, San Diego, CA), and stained for cytokines for 45 minutes at 4°C. Unless otherwise indicated, all antibodies were obtained from BD Bioscience (San Jose, CA). The antibodies used for FACS staining were APC/Cy7-anti-mouse-CD45 (clone 30-F11), PE-anti-mouse-CD3 (145-2C11), PE-anti-mouse-B220 (RA3-6B2), PE-anti-mouse-CD11b (M1/70), PE-anti-mouse-CD11c (HL3), BV785-anti-mouse-CD4 (RM4-5; BioLegend, San Diego, CA), BV786-anti-mouse-Vγ4 (UC3-10A6), PerCP/Cy5.5-anti-mouse-CD8 (53-6.7), BV421-anti-mouse-TCRγδ (GL3), BV650-anti-mouse-NK1.1 (PK136), AF647-anti-mouse-IL-17A (TC11-18H10), PE/CF594-anti-mouse-IFN-γ (XMG1.2), APC/H7-anti-human-CD45 (2D1), V450-anti-human-CD3 (UCHT1), FITC-anti-human-CD4 (RPA-T4; BioLegend, San Diego, CA), and PE-anti-human-TCRγδ (B1). Flow cytometry data were collected using the Cytoflex flow cytometry (Beckman Coulter) or Attune NxT V6 flow cytometry (ThermoFisher) and analyzed by FlowJo software (Tree Star Inc.). Lineage markers (Lin) were CD3, B220, CD11b, and CD11c.^43^

### DED Induction

To induce the DED model, adult female mice were exposed to an Intelligently Controlled Environmental System in which the relative humidity, airflow, and temperature were maintained at 15.0% to 20.0%, 2.2 ± 0.2 m/s, and 22 ± 2°C, respectively.^44^ Then, mice were subjected to subcutaneous injection of scopolamine hydrobromide (0.5 mg/0.2 mL; Sigma-Aldrich) 3 times a day for 5 days to decrease tear production. For the untreated group, mice were age and gender matched and fed in a normal environment with normal humidity (relative humidity, 60%–80%; no airflow; temperature, 21°C –23°C). Mice were sacrificed and the conjunctiva were collected and assayed for several experiments on the sixth day of this murine DED model.

### Corneal Fluorescein Staining

To determine the condition of the corneal epithelium at baseline and day 6, fluorescein was used to stain the corneas. For this, 0.5 µL of 5% fluorescein solution into the inferior conjunctiva sac using a pipette gun. Three minutes later, the corneal epithelial staining was graded using a slit-lamp microscope under a cobalt blue filter light. The degree of punctate staining was assessed in a masked fashion with a standard grading system (National Eye Institute, Bethesda, MD) of 0 to 3 for the central, superior, inferior, nasal, and temporal areas of the cornea.^45^

### Quantitative Real-Time Polymerase Chain Reaction (PCR)

Total RNA from conjunctivas was extracted according to the manufacturer's instructions (RNeasy mini kit, Qiagen, Crawley, UK). The RNA concentration was measured at 260 nm and stored at −80°C. Complementary DNA was synthesized from 0.5 µg of total RNA using random primer and M-MLV reverse transcriptase (Applied Biosystems, Paisley, UK). The sequence of the primers was: for GAPDH: sense, 5′-ATGTTCGTCATGGGTGTGAA-3′, and antisense, 5′-GGTGCTAAGCAGTTGGTGGT-3′; for IL-17A: sense, 5′- AAAGCTCAGCGTGTCCAAAC -3′, and antisense, 5′-ACGTGGAACGGTTGAGGTAG-3′; for RORγt: sense, 5′-CTGCGACTGGAGGACCTTCTAC-3′, and antisense, 5′-CAGGACGGTTGGCATTGATGAG-3′; for NLRP3: sense, 5′-CGTGAGTCCCATTAAGATGGAGT-3′, and antisense, 5′-CCCGACAGTGGATATAGAACAGA-3′; for IFN-γ: sense, 5′-TCCTCGCCAGACTCGTTTTC-3′, and antisense, 5′-GTCTTGGGTCATTGCTGGAAG-3′; for IL-1β: sense, 5′-ATGATGGCTTATTACAGTGGCAA-3′, and antisense, 5′-GTCGGAGATTCGTAGCTGGA-3′; for IL-23: sense, 5′-GCACCTGCTTGACTCTGACATC-3′, and antisense, 5′-GCTGCCACTGCTGACTAGAACT-3′. A quantitative PCR analysis was undertaken using the Power SYBR Green PCR Master Mix (Applied Biosystems) and Applied Biosystems Quant Studio 6 Real-Time PCR System (Applied Biosystems). The results were analyzed using the comparative threshold cycle method and normalized with GAPDH as an endogenous reference.

### Periodic Acid-Schiff Staining for Goblet Cells

After removal, the eyeballs together with eyelids were fixed with eyeball fixation fluid (10% formaldehyde, 40% absolute ethanol, 40% water, and 10% glacial acetic acid) for 12 to 24 hours at room temperature, and then embedded in paraffin. Serial sections with a thickness of 5 µm were cut from each sample. The sections were deparaffinized and stained with the periodic acid Schiff reagent for highlighting mucous as well as goblet cell secretion products stored in the cells. Positively stained goblet cells were counted in the conjunctiva, and the distance between the first and last goblet cells was measured. Data were presented as the average number of goblet cells per millimeter under a microscope (Leica DM750, Leica Microsystems, Wetzlar, Germany).

### Western Blot Analysis

A Western blot analysis was performed using standard techniques. In brief, conjunctivas were homogenized in RIPA lysis buffer with PMSF (RIPA: PMSF = 100: 1) (Beyotime, Shanghai, China), to extract total protein. After being quantified by a BCA protein assay kit (Beyotime), protein samples were separated using a 12% TGX Stain-Free FastCast Acrylamide Kit (Bio-Rad, Hercules, CA) and blotted to PVDF membranes (Merck-Millipore, Darmstadt, Germany). Membranes were blocked with 5% non-fatty milk and incubated with primary antibodies against GAPDH (1:5000, Cell Signaling Technology, Danvers, MA), IL-17A (1:1000, Abcam, Cambridge, UK). Membranes were then washed, incubated with appropriate peroxidase-conjugated secondary antibodies, and developed into an ECL kit (Najm Biotech ECL, Tehran, Iran).

### In Vivo Depletion of γδ T Cells in C57BL/6 Mice

The γδ T cells in Figure 5 (Clone: UC7-13D5) were depleted using antibodies from Bio X Cell. Antibodies were administered intraperitoneally (200 µg/mouse) on day 1 and day 3 after DED induction on day 0. In Vivo MAb, Polyclonal Armenian Hamster IgG (Bio X cell, BE0091) was used as an isotype control.

### Statistical Analyses

Data are expressed as mean ± standard error of the mean. The unpaired *t*-test, Mann–Whitney *U* test, and one-way ANOVA were performed for multiple comparisons among groups according to the data context. Normality was confirmed with the Shapiro–Wilk normality test. Data were analyzed on GraphPad Prism software v 7.0. *P* values were determined. A *P* value of less than 0.05 was considered statistically significant.

## Results

### γδ T Cells Are Associated With Patients With Severe DED

To investigate the tissue–resident immune cell function in DED, we set out by analyzing the immune cell compositions in human conjunctiva. We enrolled 11 patients with mild DED and 11 patients with severe DED to determine if γδ T cells were associated with the severity of DED and cornea epithelial barrier injury. The demographics and clinical features of the mild and severe DED groups are shown in Table. There were no significant differences in the mean age between the 2 groups. The mean Ocular Surface Disease Index was significantly higher in the severe DED group (*P* < 0.05), although the TBUT and Schirmer Ⅰ test results were both lower than those in the mild DED group.

**Table.** Demographics and Clinical Characteristics of Patients With Mild and Severe DED

Characteristics	Mild DED	Severe DED	*P* Value
Mean age ± SEM (range)	40.450 ± 4.603 (20–66)	49.910 ± 2.151 (38–64)	0.0775
OSDI (0–100) ± SEM	26.330 ± 4.405	48.040 ± 5.974	<0.01
TBUT(s) ± SEM	3.300 ± 1.767	2.000 ± 1.483	0.1231
Schirmer I (mm) ± SEM	10.900 ± 8.185	8.273 ± 5.293	0.6158

OSDI, Ocular Surface Disease Index (grade estimated by the Oxford scheme); SEM, standard error of the mean; TBUT, tear-film breakup time.

**Figure 1.**
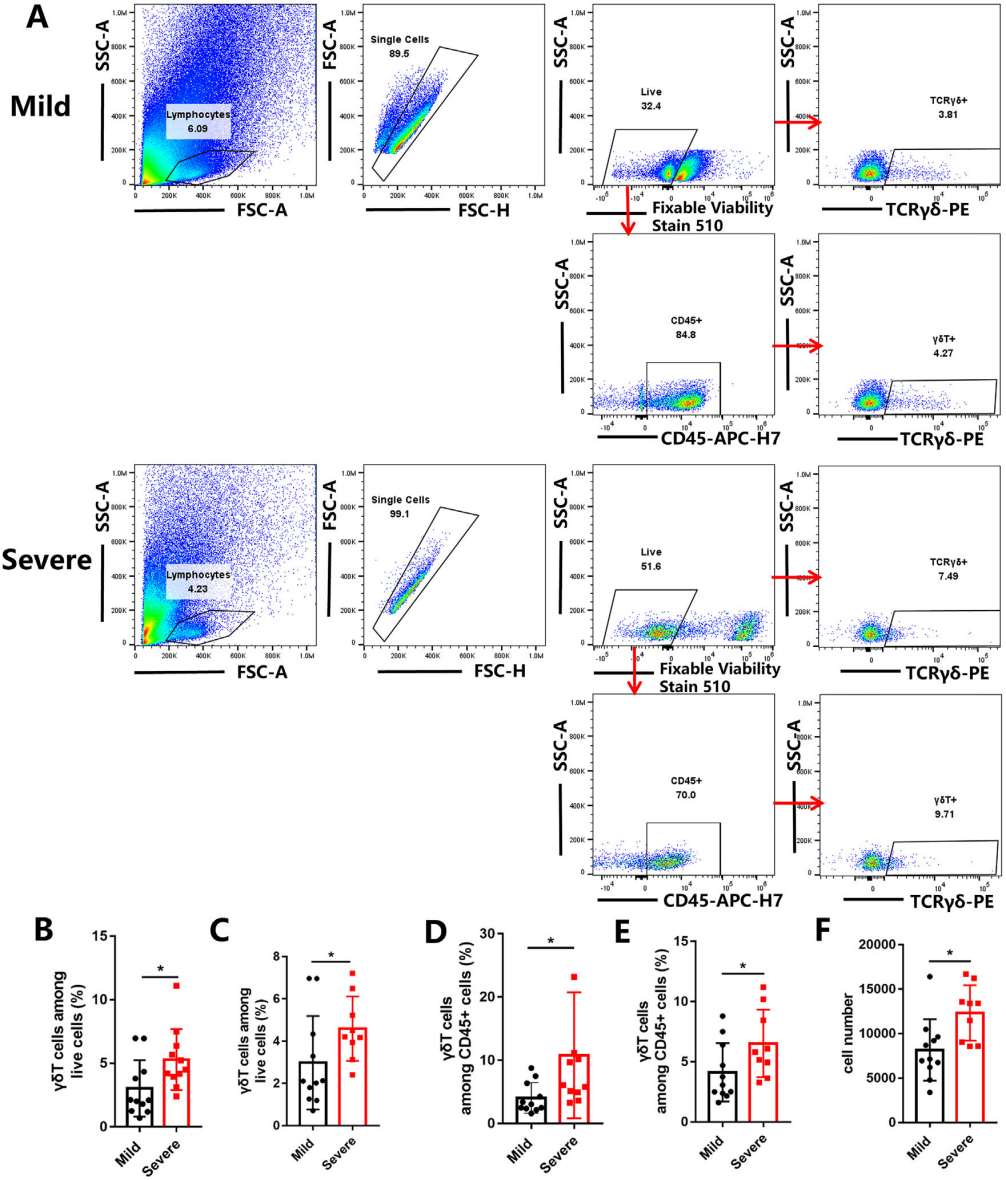
γδ T cells were identified based on positive expression of γδ T cell receptor (TCRγδ). (**A**) Gating strategy and representative flow cytometry plots for analyzing γδ T cells on the human ocular surface. The γδ T cells were gated on live cells and live CD45^+^ cells, respectively. (**B,** **C**) The quantitative summary of γδ T-cell proportion among live cells, with (**B**) or without (**C**) the 2 outliners. (**D,** **E**) The quantitative summary of γδ T-cell proportion among CD45^+^ cells, with (**D**) or without (**E**) the 2 outliners. (**F**) The quantity in the human ocular surface. In the graph, each bar represents the mean ± standard error of the mean. *P < 0.05. *n* = 11 for patients with mild DED, and *n* = 11 for patients with severe DED.

Given the most recent studies revealing the crucial roles of γδ T cells in mucosal immunity,^23^^,^^24^^,^^25^ we focused our efforts on γδ T cells in the ocular surface of patients with DED by brush cytology. The gating strategy for analyzing γδ T cells is shown in Figure 1A. The γδ T cells were gated on live cells and live CD45^+^ cells, respectively. The quantitative summary of γδ T-cell proportion was analyzed with (Figs. 1B and D) or without (Figs. 1C and 1E) 2 outliners. The percentage and number of γδ T cells in the ocular surface of patients with severe DED was significantly higher than that of patients with mild DED analyzed either way (*P* < 0.05) (Figs. 1B–1F).

### γδ T Cells in Normal Murine Conjunctiva Express High Levels and Are the Main Producers of IL-17A But Not IFN-γ

**Figure 2.**
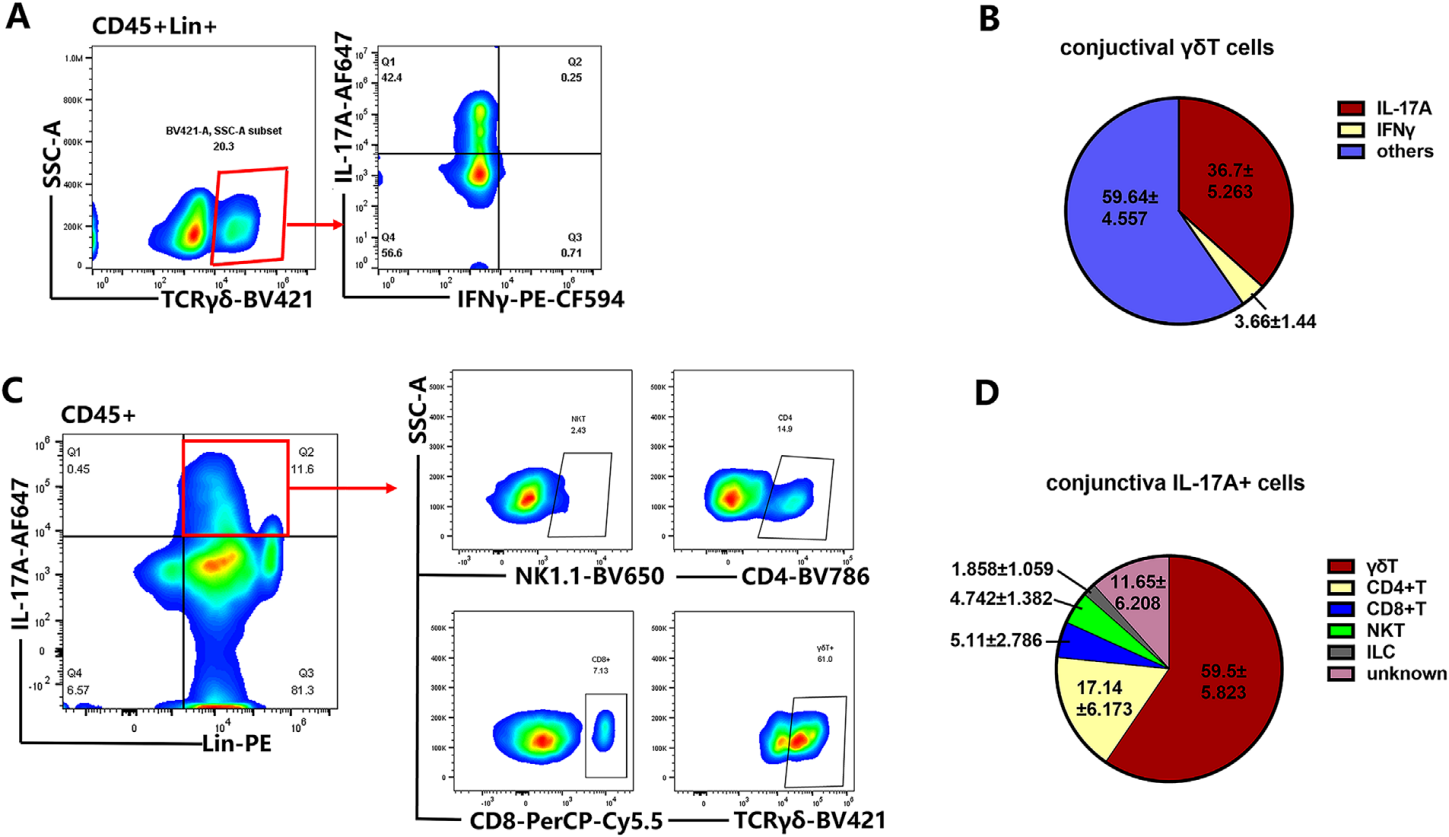
The characteristic of γδ T cells and the proportion of IL-17A–producing cells in the normal murine conjunctiva. (**A,** **B**) γδ T cells from conjunctiva expressed IL-17A predominantly and IFN-γ to a much lesser degree. The mononuclear cells from the conjunctiva were stimulated with PMA plus ionomycin in the presence of brefeldin A (BFA) for 4 hours and analyzed by FACS. The representative graph and pie chart for the expression IL-17A and IFN-γ in γδ T cells were shown (*n* = 6). (**C,** **D**) Flow plots represent IL-17A production in mononuclear cells from the murine conjunctiva after PMA and ionomycin stimulation for 4 hours in the presence of BFA. Gated on conjunctival CD45^+^ cells, the representative graph of the IL-17A–producing cells, including innate lymphoid cells (ILCs), CD4^+^ T, CD8^+^ T, γδ T, and NK T cells are shown (*n* = 8).

Previous studies in DED showed that both IFN-γ and IL-17A contribute to the corneal barrier disruption.^8^^,^^9^ These findings indicate that ocular surface infiltrating T cells in DED are Th1 and Th17 effectors, which are generated in the regional draining lymph nodes.^46^ γδ T cells can be divided into either IFN-γ–producing γδ T cells or IL-17A–producing γδ T cells.^25^^,^^26^ These results showed that γδ T cells occupied a high proportion of immune cells in the conjunctiva. Thus, we measured the proportion of γδ T-cell subsets that produce IL-17A or IFN-γ. To evaluate the expression of IL-17A and IFN-γ from γδ T cells, the mononuclear cells from conjunctiva were stimulated with PMA plus ionomycin for 4 hours in the presence of brefeldin A. The results from FACS data demonstrated that the majority of γδ T cells from conjunctiva expressed IL-17A (36.7% ± 5.263) but not IFN-γ (3.66% ± 1.44) (Figs. 2A, 2B). Further, the analysis of the total IL-17A–producing cells in the mouse conjunctiva revealed that IL-17A was produced by CD45^+^ cells, especially in Lin^+^ cells (Figs. 2A–2D). Consistently, γδ T cells accounted for 59.500% ± 5.823% of the IL-17A–producing cells in the steady state, with approximately 3.5 times more than that of CD4^+^ T cells (17.14% ± 6.173), and only a small proportion of IL-17^+^ cells were TCR-negative cells (Fig. 2D).

### IL-17A Is Elevated in the Ocular Surface in a Mouse DED Model

**Figure 3.**
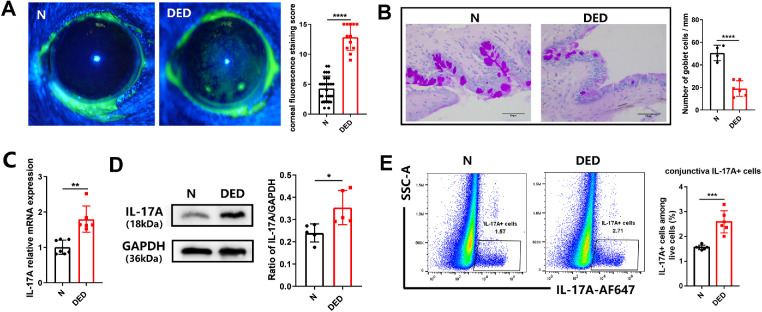
IL-17A is elevated in the ocular surface of the mouse DED model. (**A**) Representative image of corneal fluorescein staining (*n* = 10) and changes in the corneal fluorescein staining scores. (**B**) Conjunctival tissue sections were stained with periodic acid Schiff (PAS) and the goblet cells were counted. (**C,** **D**) Quantitative PCR and immunoblot for the expression of IL-17A in the conjunctiva of the control group and the DED group. (**E**) Representative FACS plots and summary of live IL-17A^+^ cells in the conjunctiva. In the graph, each bar represents the mean ± standard error of the mean. *P < 0.05, **P < 0.01, ***P < 0.001, ****P < 0.0001.

Previous results have shown that the DED model recapitulates several features of DED, including corneal staining and conjunctival goblet cell loss.^44^^,^^47^ We used low humidity and increased airflow with systemic injection of scopolamine to induce murine DED. After 5 days of scopolamine and Intelligently Controlled Environmental System exposure, the mouse corneal fluorescein staining scores increased significantly in the DED group (*P* < 0.0001), which indicated the corneal epithelial defects (Fig. 3A). Goblet cells were counted manually from the conjunctiva staining. In the control group of mice, the mean ± standard error of mean number of goblet cells was 50.5 ± 3.428/mm (Fig. 3B). The DED group demonstrated drastic decreases in conjunctival goblet cells for only 19.0 ± 2.781/mm (Fig. 3B). The corneal fluorescein staining scores and conjunctival goblet cell quantification together verified the pathology of the DED animal model.

**Figure 4.**
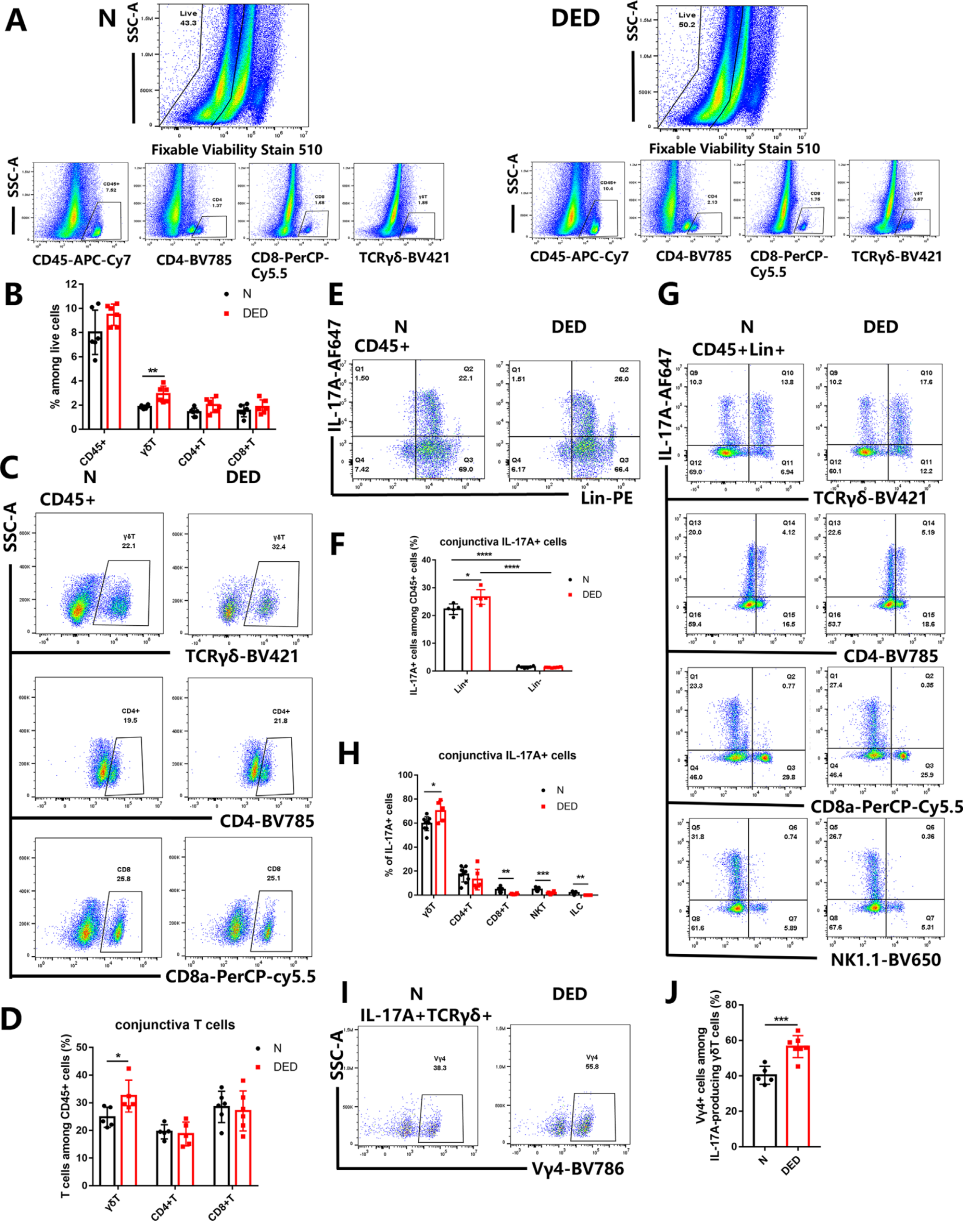
The γδ T cells are the main producers of IL-17A in the DED mouse conjunctiva. (**A,** **B**) Representative flow cytometry plots and quantitative summary of CD45^+^ cells, γδ T cells, CD4^+^ T cells, and CD8^+^ T-cell percentage among the conjunctiva live cells. (**C,** **D**) Representative flow cytometry plots and quantitative summary of γδ T cells, CD4^+^ T cells, and CD8^+^ T cells in the conjunctiva of the mice in the control group (N) and the DED group. (**E–H**) The conjunctival cells were stimulated with PMA plus ionomycin for 4 hours in the presence of brefeldin A (BFA). The cells were stained with mAb against surface markers of each T cell subset and innate lymphoid cells, then intracellularly with anti–IL-17A mAb and analyzed by flow cytometry. Representative flow cytometry plots and proportions of IL-17A–producing ILCs among CD45^+^ lymphoid cells are shown in (**E**) and (**F**). IL-17A^+^Lin^+^ cell populations from conjunctiva were further analyzed for TCRγδ, CD4, CD8a, and NK1.1 expression by flow cytometry (**G**). The proportion of IL-17A–producing cell subsets (γδ T, CD4^+^ T, CD8^+^ T, NK T cells, and ILCs cells) among CD45^+^ cells in the control and DED groups are shown in (**H**). (**I,** **J**) The representative FACS plots and summary of the Vγ4 subset of IL-17A–producing γδT cells in the conjunctiva. Data represent the mean ± standard error of the mean (*n* = 10–12). *P < 0.05, **P < 0.01, ***P < 0.001, ****P < 0.0001.

Previous findings demonstrated that the transcript level of IL-17A and the number of IL-17A^+^ cells assessed by immunofluorescence in the ocular surface of DED mice are increased.^7^^,^^9^ We first examined the production of IL-17A in the mouse conjunctiva by quantitative PCR, immunoblot, and FACS. Consistently, IL-17A mRNA and protein levels were significantly increased in DED mouse conjunctiva (Figs. 3C, 3D). By intracellular staining of IL-17A, we found the percentage of IL-17A^+^ cells among all the conjunctiva live cells were up-regulated in DED mouse conjunctiva (from 3.424% to 5.878%) (Fig. 3E). Next, we examined the cellular source of IL-17A in the mouse conjunctiva. We focused on IL-17A production by γδ T cells, CD4^+^ T cells, and CD8^+^ T cells based on results obtained in Figure 3. We analyzed CD45^+^ cells, γδ T cells, CD4^+^ T cells, and CD8^+^ T cells among the conjunctival live cells (Fig. 4A). No statistical difference was observed in CD45^+^ cells, CD4^+^ T cells, or CD8^+^ T cells between the control group and the DED group (Fig. 4B). The percentage of γδ T cells among the conjunctival live cells increased from 1.8470% ± 0.0627% in the control group to 2.9050% ± 0.2738% in the DED group. Further, T cells were gated on CD45^+^ cells. The proportion of γδ T cells among CD45^+^ cells increased from 24.76% ± 3.64% in the control group to 32.440% ± 5.762% in the DED group. In contrast, there was no significant change in the proportion of conjunctiva CD4^+^ T cells and CD8^+^ T cells (Figs. 4C, 4D). We next performed intracellular staining to investigate the IL-17A–producing cells in the conjunctiva. Lin^+^ cells appeared to be the major (90%) producers of IL-17A in the mouse conjunctiva, and the proportion of these cells increased in the DED group (Figs. 4E, 4F). Within Lin^+^ cells, CD8^+^ T cells, NK T cells, and ILCs (including ILC2 and ILC3) produced negligible levels of IL-17A. The main source of IL-17A–producing cells was γδT cells and their proportion increased in DED mice (Figs. 4G, 4H). We further analyzed the Vγ4 subset of IL-17A–producing γδ T cells, the IL-17A^+^ Vγ4 subset increased significantly in DED mouse conjunctiva, from 40.340% ± 2.263% to 56.530% ± 2.321% (Figs. 4I, 4J).

### γδ T-Cell Deficiency Decreases the Susceptibility of Mice to DED

**Figure 5.**
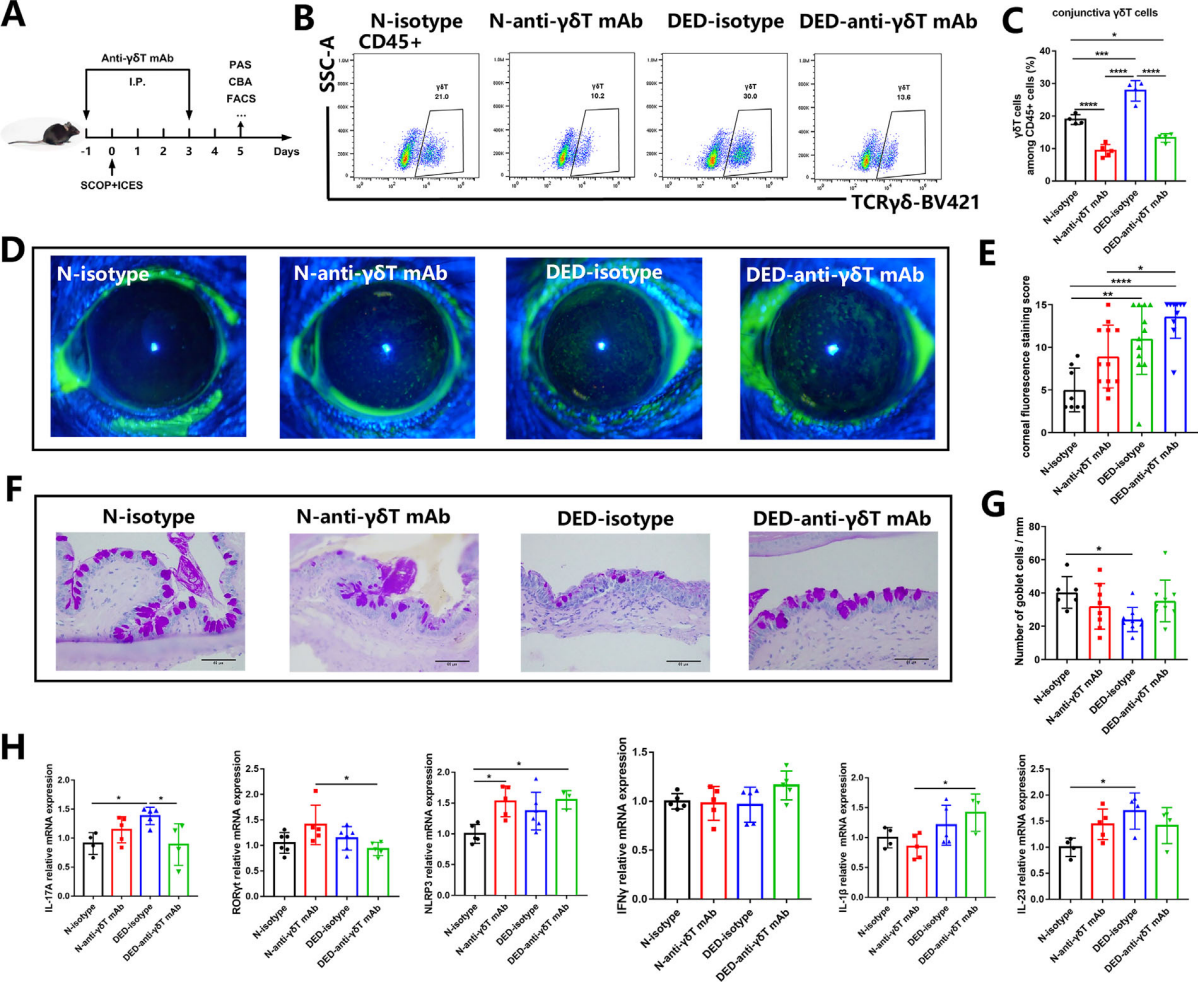
Depletion of γδ T cells significantly decreases the severity of DED inflammation. (**A**) WT mice were administrated intraperitoneally with anti-γδ T-specific mAb or isotype control Ab 1 day before and 3 days after DED induction. (**B,** **C**) The depletion efficiency in mouse conjunctiva was confirmed on day 5 by flow cytometry (*n* = 3–5). (**D**) Representative images of corneal fluorescein staining (*n* = 10). (**E**) Changes in the corneal fluorescein staining scores. (**F**) Conjunctival tissue sections were stained with periodic acid Schiff (PAS) and the goblet cells were counted (**G**). (**H**) Quantitative PCR analysis for the expression of IL-17A, RORγt, NLRP3, IFN-γ, IL-1β, and IL-23 in the mouse conjunctiva. In the graph, each bar represents the mean ± standard error of the mean. *P < 0.05, **P < 0.01, ***P < 0.001, ****P < 0.0001.

These results showed that γδ T cells, especially IL-17A^+^ γδ T cells, altered consistently with the transcript and protein levels of IL-17A in the mouse conjunctiva (Figs. 3, 4). To further determine the roles of γδ T cells in the DED, we used neutralizing antibodies and TCRδ-deficient (*TCRδ^−^^/^^−^*) mice to ablate γδ T cells in mice. Two doses of anti-γδ T mAb injection were adopted 1 day before and 3 days after DED treatment based on the manufacturer's instructions as previously reported (Fig. 5A).^23^ We examined the depletion efficiency in the mouse conjunctiva at days 0, 2, and 5 after the anti-γδ T mAb injection. Injection of the mAb resulted in the depletion of γδ T cells to 68.52%, 40.41%, and 50.57% among CD45^+^ cells at days 0, 2, and 5, respectively (Supplementary Fig. S2). Moreover, we confirmed that both in normal and DED groups, mAb injection led to the depletion of γδ T cells up to 50% (Figs. 5B, 5C). As compared with isotype controls, there were no significant differences in corneal fluorescein staining scores for the anti-γδ T mAb treatment group, either in the control or DED mice (Figs. 5D, 5E). However, the conjunctival goblet cell density increased slightly in the DED group (Figs. 5F, 5G), accompanied by the decreased conjunctiva mRNA levels of IL-17A, RORγt, and IL-23 (*P* < 0.05) (Fig. 5H). RORγt is a transcription factor required for IL-17 induction.^48^ These data indicate that γδ T cells may promote the severity of DED through increasing inflammatory program.

**Figure 6.**
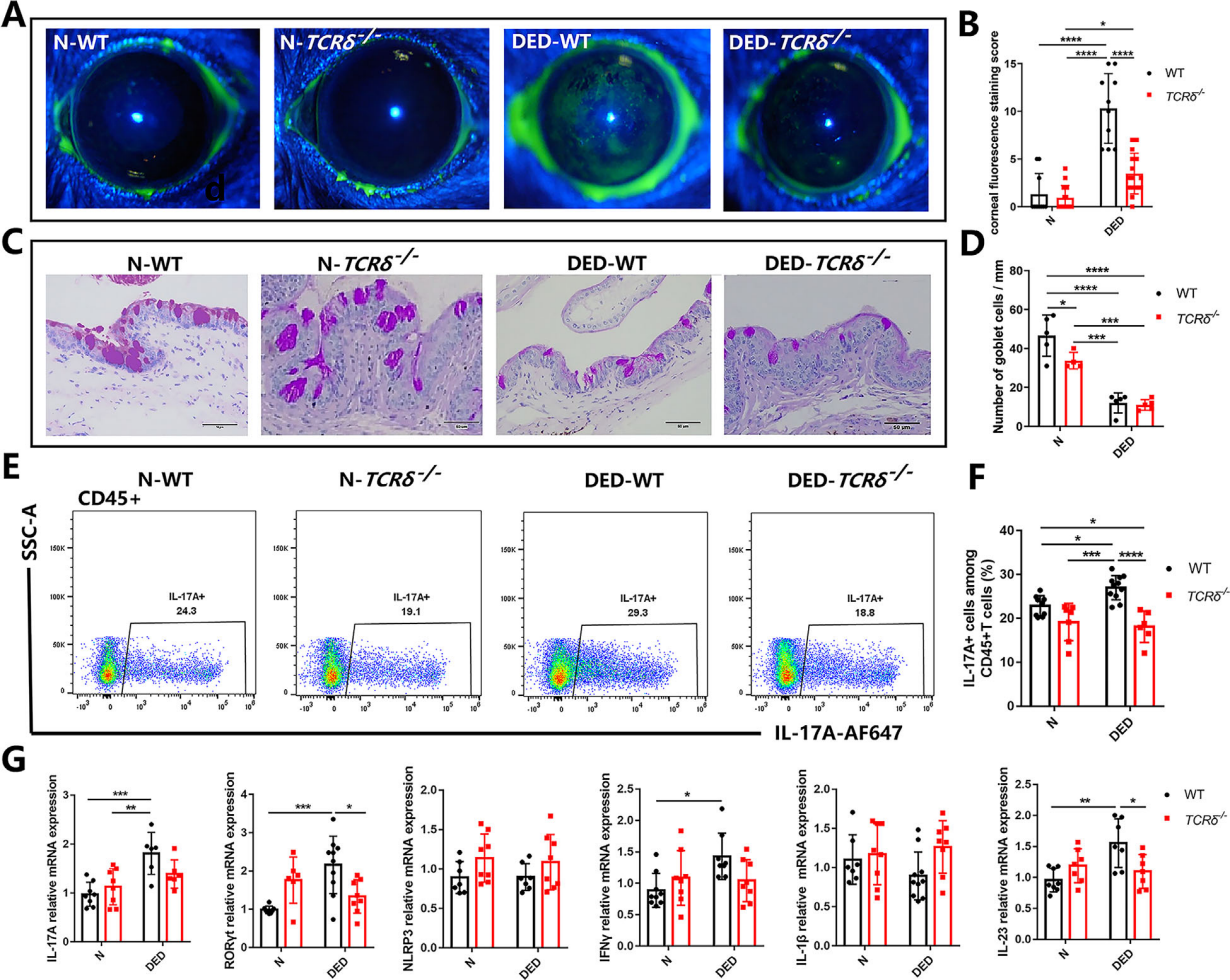
γδ T cells are pathogenic in DED inflammation and ocular damage. (**A**) Representative images of corneal fluorescein staining (*n* = 10). (**B**) Changes in the corneal fluorescein staining scores. (**C,** **D**) Conjunctival tissue sections were stained with periodic acid Schiff (PAS) and the goblet cells were counted (**D**). (**E,** **F**) Representative FACS plots and summary of IL-17A^+^CD45^+^ lymphoid cells in the conjunctiva. (**G**) Quantitative PCR analysis for the expression of IL-17A, RORγt, NLRP3, IFN-γ, IL-1β, and IL-23 in the mouse conjunctiva. In the graph, each bar represents the mean ± standard error of the mean. *P < 0.05, **P < 0.01, ***P < 0.001, ****P < 0.0001.

Next, we acquired the *TCRδ^−^^/^^−^* mice and investigated the DED pathology between WT and *TCRδ^−^^/^^−^* mice. We observed no obvious differences between *TCRδ^−^^/^^−^* mice and WT mice in steady state. In comparison with WT mice, *TCRδ^−^^/^^−^* mice had a significantly lower fluorescein staining score (*P* < 0.0001) after DED induction, suggesting a less severe DED disease, although the number of goblet cells seemed to have no obvious difference. After DED induction, *TCRδ^−^^/^^−^* mice showed an average of 8.909% decrease in IL-7A–producing cells in the conjunctiva compared with those of WT mice (Figs. 6E, 6F). Next, we detected the gene expression of inflammatory cytokines in the conjunctiva. The levels of IL-17A, RORγt, IFN-γ, and IL-23 were decreased in *TCRδ^−^^/^^−^* mice compared with the WT group after DED induction (Fig. 6G).

## Discussion

Our studies evaluated human and experimental murine DED for evidence of γδ T-cell–mediated inflammation. We assessed the proportion of γδ T cells on the ocular surface of patients with mild and severe DED. The main clinical difference between the 2 groups was the degree of corneal epithelial defect. We found that the percentage and collected number of γδ T cells in patients with severe DED was higher than that of patients with mild DED (Fig. 1). These results were corroborated in the mouse DED model.

The contribution of various immune subsets with their associated cytokines to the DED initiation and progression has gained attention recently, but remains unresolved. The presence of CD4^+^ T cells at the ocular surface in DED^49^ and the successful treatment of ocular surface inflammation with topical cyclosporine suggested a potential role for adaptive immunity in DED in the previous studies.^50^ Initiating an adaptive immune response requires that antigens at the site of inflammation are processed and presented by professional APCs to activate and expand antigen-specific effector T cells. However, the antigen or antigens that initiate this response in DED has not been identified.^51^ Differing from αβ T cells, γδ T cells do not recognize classical peptide antigens, their TCRs are non–major histocompatibility complex restricted, and they can also respond to pathogen-associated cytokines and molecular patterns without TCR ligands. Additionally, they recognize stress-induced self-molecules, which indicate infection and cellular transformation.^52^ In our study, we found that γδ T cells in cervical lymph nodes were much sparser than in conjunctiva. Conversely, the γδ T cells constitute a higher proportion than CD4^+^ T cells among CD3^+^ T cells in mouse conjunctiva (Supplementary Fig. S1), consistent with previous studies showing that the majority of intraepithelial lymphocytes (IELs) in the murine conjunctiva were γδ T cells (up to 50%).^22^ Considering that γδ T cells are tissue-resident cells and act as the first line of defense in DED, we tend to think that the increased γδ T cells come from proliferation rather than from circulation. Adopting a mouse model to induce an early stage DED,^53^^,^^54^ we have here provided evidence that γδ T cells contribute significantly to the inflammatory response during the induction phase of DED.

The proinflammatory role of IL-17A in autoimmune diseases has drawn much attention over the past decades.^9^^,^^23^^,^^55^ IL-17A increases in the ocular surface of patients with DED and animal models.^7^^,^^9^^–^^13^ The expression of IL-17RA by the corneal and conjunctival epithelia further supports the importance of an IL-17A–mediated cytokine cascade in the immunopathogenesis of DED.^7^ We have validated that the transcript and protein levels of IL-17A as well as IL-17A–expressing cells all significantly increased in DED mouse conjunctiva (Fig. 3). Moreover, we found that after desiccating stress on the ocular surface, γδ T cells in conjunctiva expressed significantly higher levels of IL-17A, approximately 3 times more than Th17 cells (Fig. 4). Interestingly, 2 independent studies reported in 2009 showed that IL-17A is associated with disruption of corneal epithelial barrier function: CS De Paiva et al. found the number of IL-17A–producing cells increased in the DED mouse cornea and conjunctiva, although no evidence was provided to reveal the identity of IL-17A–producing cells^9^; Chauhan et al^7^ focused on the changes of Th17 cells in cervical lymph nodes and found the mRNA levels of IL-17A increased in DED mouse conjunctiva. Further, Chauhan et al were able to show that DED inflammation was lower in mice treated with anti-IL–17A antibodies.^7^^,^^56^ Almost at the same time, Alam et al^39^ found that the Pinkie mouse strain with a loss-of-function RXRα mutation has increased signs of DED and a 4-fold greater percentage of conjunctival γδ T cells with a higher expression of IL-17 under homeostatic conditions. Notably, disruption of the IL-17A signal has been associated with the development and progression of various autoimmune or autoinflammatory diseases, such as rheumatic diseases.^57^ Importantly, the biological therapies targeting IL-17A have been approved to treat various diseases.^58^ It is therefore highly promising to repurpose some of the strategies for IL-17A blockage in the treatment of DED. Moreover, the delineation of the pathological cell types with the associated pathological factors may inform the development of low-cost small molecule target drugs as well.

In our study, we unexpectedly found that normal mice with anti-γδ T mAb treatment exhibited higher fluorescein staining scores, a lower number of goblet cells, and higher conjunctiva mRNA levels of IL-17A, RORγt, and NLRP3, as well as IL-23 than the isotype group (Fig. 5). This observation implicates a trend of the impaired ocular surface in normal mice. Thus far, we have not been able to fully understand the underlying mechanism, but one possibility is that low levels of IL-17A may contribute to maintaining ocular surface homeostasis under physiological conditions.^59^ In the meantime, the number of goblet cells in the conjunctiva of DED mice did not seem to improve significantly after partial or complete elimination of γδ T cells. However, mice lacking γδ T cells had lower fluorescein staining scores (*P* < 0.0001) after DED induction (Figs. 5 and 6). In contrast, patients with DED with a severe cornea epithelial barrier injury have a high percentage of γδ T cells on the ocular surface. It is likely that γδ T cells on the ocular surface play diverse roles in epithelial wound repair and immunoregulation. This dual function mode also arises in the gut epithelium, and studies have shown that γδ IELs contribute to both the progression of immune-mediated colitis^60^ and epithelial restitution following an injury during the inflammatory response.^61^ We thus speculate that the effects on goblet cells and pathological scores might reflect the different aspects of γδ T-cell functions.

The conjunctiva is a thin mucous membrane from the eyelid margin to the corneoscleral limbus. It forms a physical protective barrier that prevents the entrance of extraneous matter into the ocular globe. As a mucosal surface, the conjunctiva is equipped with a diverse array of innate defense mechanisms, such as antimicrobial peptides, macrophages, mast cells, and neutrophils that prevent pathogen invasion and maintain mucosal integrity. And it also harbors specific immune cells, including T and B cells, as in other mucosal epithelia.^15^^,^^16^ The conjunctiva, like other mucosal tissues, is covered by epithelium that contains multiple IELs. The superficial layer of conjunctiva cells, containing IELs, has often been studied with techniques such as brush technology and impression cytology.^62^^,^^63^ And the ocular surface cytology has been used to evaluate many ocular surface diseases, including keratoconjunctivitis sicca, vernal keratoconjunctivitis, keratoconus, and so on.^64^^–^^66^ We use brush technology to obtain the ocular surface cytology sample and analyze the IELs by flow cytometry. A major challenge with this technique is the relatively few immune cells obtained. The total number of CD45^+^ cells per individual varied from 800 to 5000 cells, similar to what has been previously reported.^62^ Owing to the limited cell numbers, we have selected the surface marker to analyze the proportion of γδ T cells. Future efforts are, therefore, warranted to analyze the production of IL-17A and IFN-γ in a more comprehensive manner.

The limitations of this study include the use of a DED animal model in the acute induction period that may not well establish a full-blown adaptive immunity.^67^ Future work is warranted to investigate whether the γδ T cells participate in all stages of DED, especially the chronic phase comparable to patients with DED in the clinical setting. In addition, γδ T cells may interact with CD4^+^ T cells locally or in the draining lymph nodes to mediate the different stages of DED.

In summary, our work provides evidence that the tissue-resident γδ T cells are enriched in the conjunctiva and express IL-17A but not IFN-γ, and play a critical role in exacerbating DED. Our study provides insights into the immune pathogenesis of DED from the perspective of both immune cell subtypes and pathological cytokines. The findings here have enhanced our knowledge of developing therapeutic strategies for DED.

## Supplementary Material

Supplement 1
