# Supplementary material for: Conjunctiva Resident γδ T Cells Expressed High Level of IL-17A and Promoted the Severity of Dry Eye
Source: Invest Ophthalmol Vis Sci. 2022 Nov 9;63(12):13. doi: 10.1167/iovs.63.12.13 (PMC9652718; doi:10.1167/iovs.63.12.13)
Supplement: Supplement 1 [file iovs-63-12-13_s001.pdf]

# Supplementary Information

# Conjunctiva Resident γδ T Cells Expressed High Level of IL-17A and Promoted the Severity of Dry Eye

Ling Li[#1,2], Yanxiao Li[#1], Xinhao Zhu[1], Zihao Liu[1], Zhuo Tang[1], Biao Wu[3], Han Wen[1], Jianshu Yuan[2], Qinxiang Zheng[✉1,2] and Wei Chen[✉1,2]

[1]School of Ophthalmology and Optometry and Eye Hospital, Wenzhou Medical University, Wenzhou, Zhejiang, China. [2]The Affiliated Ningbo Eye Hospital of Wenzhou Medical University, Ningbo, Zhejiang, China. [3]Shaoxing people's hospital, Shaoxing, Zhejiang, China.

[#]These authors contributed equally: Ling Li, Yanxiao Li.

✉Correspondence: Wei Chen (chenweimd@wmu.edu.cn) or Qinxiang Zheng (zhengqinxiang@aliyun.com)

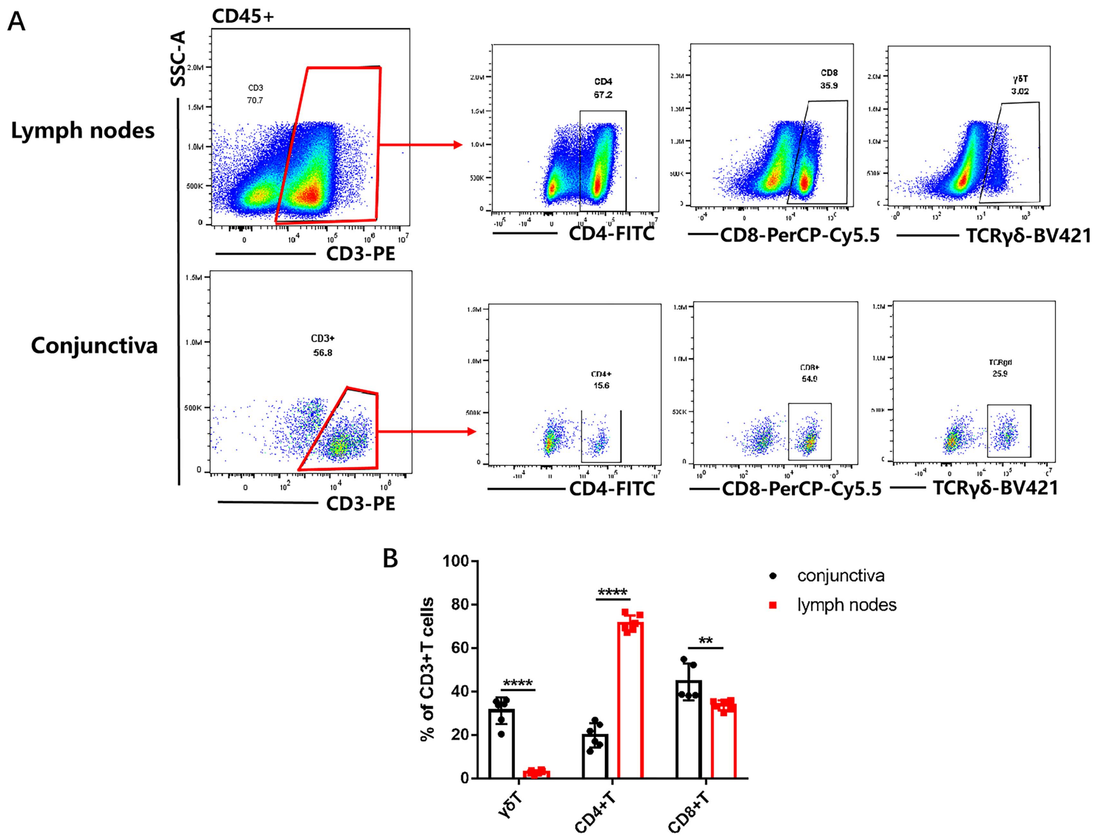


Figure S1. γδ T cells are enriched in murine conjunctiva compared to cervical lymph nodes. (A) Flow cytometry analysis of freshly isolated cells from the cervical lymph nodes and conjunctiva (n = 6 per group) stained with CD45-APC-Cy7, CD3-PE, CD4-FITC, CD8-PerCP-Cy5.5, TCRγδ-BV421 conjugated antibodies. Lymphocytes were gated based on characteristic light-scatter properties, T cells were separated by CD45$^{+}$CD3$^{+}$ and then further delineated by CD4, CD8, and TCRγδ. Numbers in the quadrants and the bar chart indicate the proportion of CD4$^{+}$T, CD8$^{+}$T, and γδ T cells in total CD3$^{+}$T cells. (B) The statistical analyses for the percentages of CD4$^{+}$T, CD8$^{+}$T, and γδ T cells in total CD3$^{+}$T cells in the conjunctiva and cervical lymph nodes were shown. In the graph, each bar represents the mean ± SEM. $^{**}p<0.01$, $^{****}p<0.0001$.

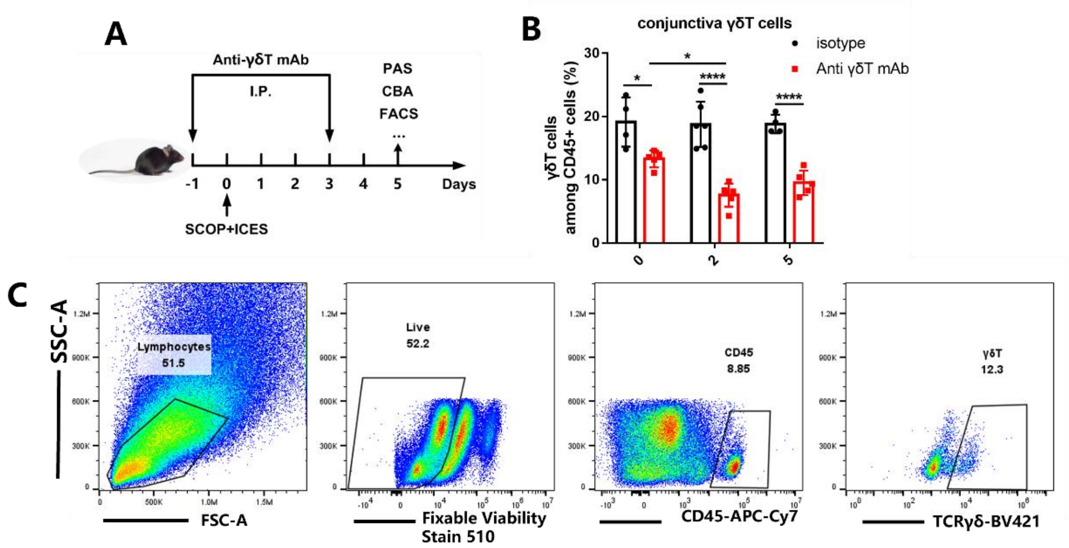


Figure S2. The depletion efficiency of the conjunctiva γδ T cells by in vivo anti-γδ T mAb. (A) A schematic drawing shows the experimental design. WT mice were administered intraperitoneally with anti-γδ T-specific mAb or isotype control Ab 1 day before and 3 days after DED induction. (B) The quantitative summary of γδ T cells proportion among live $CD45^+$ cells at day 0, 2, and 5 as the schematic drawing shows. (C) The gating strategies and representative flow cytometry plots for analyzing γδ T cells on the mice conjunctiva. In the graph, each bar represents the mean ± SEM. *p＜0.05, ****p＜0.0001.
